# Molecular Fingerprinting and Hybridity Authentication in Cowpea Using Single Nucleotide Polymorphism Based Kompetitive Allele-Specific PCR Assay

**DOI:** 10.3389/fpls.2021.734117

**Published:** 2021-10-05

**Authors:** Patrick Obia Ongom, Christian Fatokun, Abou Togola, Stella Salvo, Oluwaseye Gideon Oyebode, Mansur Sani Ahmad, Ishaya Daniel Jockson, Garba Bala, Ousmane Boukar

**Affiliations:** ^1^International Institute of Tropical Agriculture (IITA), Kano, Nigeria; ^2^International Institute of Tropical Agriculture (IITA), Ibadan, Nigeria; ^3^Bayer Research and Development Services LLC, Chesterfield, MO, United States

**Keywords:** cowpea (*Vigna unguiculata*), fingerprinting, hybridity test, QC markers, KASP assay, true F_1_ progeny, genetic distance

## Abstract

Optimization of a breeding program for increased genetic gain requires quality assurance (QA) and quality control (QC) at key phases of the breeding process. One vital phase in a breeding program that requires QC and QA is the choice of parents and successful hybridizations to combine parental attributes and create variations. The objective of this study was to determine parental diversity and confirm hybridity of cowpea F_1_ progenies using KASP (Kompetitive Allele-Specific PCR)-based single nucleotide polymorphism (SNP) markers. A total of 1,436 F_1_ plants were derived from crossing 220 cowpea breeding lines and landraces to 2 elite sister lines IT99K-573-1-1 and IT99K-573-2-1 as male parents, constituting 225 cross combinations. The progenies and the parents were genotyped with 17 QC SNP markers *via* high-throughput KASP genotyping assay. The QC markers differentiated the parents with mean efficiency of 37.90% and a range of 3.4–82.8%, revealing unique fingerprints of the parents. Neighbor-Joining cladogram divided the 222 parents into 3 clusters. Genetic distances between parents ranged from 0 to 3.74 with a mean of 2.41. Principal component analysis (PCA) depicted a considerable overlap between parents and F_1_ progenies with more scatters among parents than the F_1_s. The differentiation among parents and F_1_s was best contributed to by 82% of the markers. As expected, parents and F_1_s showed a significant contrast in proportion of heterozygous individuals, with mean values of 0.02 and 0.32, respectively. KASP markers detected true hybridity with 100% success rate in 72% of the populations. Overall, 79% of the putative F_1_ plants were true hybrids, 14% were selfed plants, and 7% were undetermined due to missing data and lack of marker polymorphism between parents. The study demonstrated an effective application of KASP-based SNP assay in fingerprinting, confirmation of hybridity, and early detection of false F_1_ plants. The results further uncovered the need to deploy markers as a QC step in a breeding program.

## Introduction

Cowpea [*Vigna unguiculata* (L.) Walp.] is a predominantly self-pollinated crop and a diploid (2n = 2x = 22) with genome size of 640.6 Mbp (Lonardi et al., 2019). Cowpea is one of the most important legume crops cultivated and consumed in dry areas of the tropics and subtropics (Boukar et al., 2016). The crop plays vital roles as a nutritional and food security crop and generates income for farmers and food vendors. On average, cowpea grains contain 25% protein, 53.2 mg/kg iron, 38.1 mg/kg zinc, 826 mg/kg calcium, 1,915 mg/kg magnesium, 14,890 mg/kg potassium, and 5,055 mg/kg phosphorus (Boukar et al., 2011). The crop is also an important source of nutritious fodder for ruminants mainly in the Sahel regions of West and Central Africa (Boukar et al., 2016, 2019). Global annual cowpea production is estimated at 6.5 million metric tons on about 14.5 million hectares (Boukar et al., 2016; Kebede and Bekeko, 2020). Of the total world production, about 83% is obtained in Africa, with West Africa accounting for 80% of African production, and Nigeria taking the lead in world production and consumption. Despite the importance of cowpea, its productivity is still below expectation in most African countries due to attack by multiple insect pests, diseases and parasitic weeds, as well as drought, heat, and low soil fertility (Carsky et al., 2001; Agbicodo et al., 2009; Boukar et al., 2016, 2018; Togola et al., 2017, 2020; Ongom et al., 2021). These constraints, if not mitigated by crop improvement through plant breeding, will continually dwindle the future of cowpea, denying farmers and consumers the opportunities to reap the potential benefits of agricultural productivity and food and nutrition security from this crop. Among multiple mitigation measures that have been advanced to curve the highlighted challenges in cowpea production, the deployment of cultivars that display resistance and/or tolerance to the stresses in farmers’ fields has been the focus of breeding programs in many research institutions.

Conventionally, the development of an improved cultivar involves an initial step in hybridization among selected parental lines having attributes of interest such as higher yield, resistance to any of the stress causing factors. This is then followed by advancing the progenies through repeated self-pollination until the lines are homozygous and stable before extensive testing are conducted to identify improved lines (Khanna, 2018; Osei et al., 2020). This process is lengthy and costly, yet the eventual creation of the intended variation through hybridization and the ability to further study the genetics of cowpea cultivar development requires validity of the initial cross. First, the parents of a cross should not be genetically similar in order to maximize the diversity for selection and improvement (Mafakheri et al., 2017; Nkhoma et al., 2020). It is therefore advantageous to fingerprint parental lines and assess relatedness prior to hybridization. Secondly, the progenies being advanced must be a product of successful cross pollination so as to capture the intended inheritance of trait combinations while maintaining an optimal level of diversity in the breeding program. Cowpea, being a highly self-pollinated crop, is inherently and botanically designed to avoid outcrossing. Consequently, hybridization requires special techniques to remove the anthers just before selfing occurs (Myers, 1996). Therefore, inaccurate timing of flower emasculation may result in self- pollinated plants and not true F_1_ hybrids as the breeders intended. This quickly becomes a quality concern in breeding programs that often produce thousands of crosses. A routine best practice in modern breeding programs incorporates quality control (QC) steps by leveraging molecular breeding tools at stages in the breeding workflow that optimize the cost benefit of delivering high quality cultivars. It has been observed that crossing in cowpea by hand emasculation is relatively easy compared to some other legumes given that its flowers are large and easy to manipulate, the keel is straight, beaked and not twisted. However, the success rate is reportedly as low as 10–20% (Myers, 1996). This low success rate is attributable to high flower abscission due to mechanical handling during emasculation (Myers, 1996). Interestingly, out of the perceived successful crosses, some end up as self-pollinated plants having escaped the manual cross-pollination process. Without any quick detection technique, these escapes would not be easily detectable, constituting in potential wasted time and resources that would be carried forward in the breeding program. Efficient and optimized breeding programs include routine QC to eliminate the false F_1_ plants early enough to make the most impact with the resources available (PASSeL, 2019). With the advancements in genetics, molecular biology and tissue culture, plant breeding is now increasingly being carried out using molecular genetic tools (Tester and Langridge, 2010; Boukar et al., 2018, 2020; Khanna, 2018). Parental fingerprinting and hybridity tests, which are among the key aspects of QC in the breeding process can now be conducted using molecular markers (Weerasinghe et al., 2004; Wu et al., 2010; PASSeL, 2019; Osei et al., 2020). The past few decades have witnessed notable progresses in the evolution of molecular marker systems and the detection platforms (Mammadov et al., 2012). Of all the marker systems, single nucleotide polymorphism (SNP) has become a standard marker type of choice in molecular breeding and genetics due to its specificity and abundance in the genome coupled with ease of automation and high-throughput detection system (Mammadov et al., 2012; Jiang, 2013). The wide-scale use and application of SNP markers in molecular breeding has been enabled by the development of user-friendly tools and platforms (Jiang, 2013). For greater precision and effectiveness in marker assisted selection (MAS), multiplex detection system for SNPs has been developed (Jiang, 2013; Bernardo et al., 2015; Nadeem et al., 2018). KASP, developed by Laboratory of the Government Chemist (LGC) Genomics (Braae et al., 2014) is an example of a recently developed multiplexed SNP marker detection system that combines several markers in a single assay (Nadeem et al., 2018). It is a standardized technology whereby genotyping is based on fluorescence. The technique rests on an allele-specific oligo extension and fluorescence resonance energy transfer that are used for signal detection (Braae et al., 2014; Nadeem et al., 2018). The KASP assay, together with other types of markers, have been applied successfully for germplasm diversity analysis or fingerprinting and hybrid purity tests in several crops including tomato (Osei et al., 2020), melon (Kishor et al., 2020), lettuce (Patella et al., 2019a), rice (Yashitola et al., 2002; Sundaram et al., 2008), maize (Patella et al., 2019a), and cotton (Selvakumar et al., 2010).

Recently in cowpea, there has been tremendous efforts to develop genomic resources to facilitate molecular-aided breeding. In this regard, Muchero et al. (2009a) developed an Illumina 1,536-SNP GoldenGate genotyping array for cowpea derived from 183,118 expressed sequence tags (ESTs). Out of these, 928 quality SNPs were incorporated into a consensus genetic map of cowpea, developed based on 741 recombinant inbred lines derived from 6 mapping populations. This linkage map spans 680 cm with 11 linkage groups and an average marker distance of 0.73 cm. This linkage map provides a framework for molecular marker deployment in cowpea breeding. In addition, a whole-genome shotgun (WGS) assembly and a bacterial artificial chromosome (BAC) physical map were developed from the IITA cowpea variety IT97K−499−35 (Muñoz-Amatriaín et al., 2017). These genomic resources allowed the development of 51,128 Illumina Cowpea iSelect Consortium Array (Muñoz-Amatriaín et al., 2017). Several of these SNPs were utilized to develop KASP markers and are available either as trait-linked markers for marker-assisted breeding or for QA/QC system (Huynh et al., 2015; Boukar et al., 2016, 2018). A database of available KASP markers for QC in cowpea is obtainable from Excellence in Breeding platform at https://excellenceinbreeding.org/module3/kasp. However, the effectiveness of these QC markers for large scale detection of F_1_ hybridity in cowpea and the rate of success in obtaining true F_1_s in highly productive breeding programs are not well-documented. The objective of the present study was to evaluate the genetic relationships among cowpea parental lines and determine the hybridity of resultant putative F_1_ progenies for further advancement in the program using KASP-based SNP assay. The study highlights the significance of molecular marker deployment for QC in cowpea breeding and corroborates the efforts toward optimization of the breeding program for increased genetic gain.

## Materials and Methods

### Plant Materials

Parental lines consisted of 89 lines recycled from Initial Evaluation Trial (IET), 31 lines from Preliminary Yield Trials (PYT), 85 from Advanced Yield Trials (AYT), and 15 landraces, making a total of 220 female parents (Table 1). These were crossed with two male sister lines (IT99K-573-1-1 and IT99K-573-2-1) developed by IITA and are known to be sources of striga resistance. The line IT99K-573-2-1 was used as a male for 15 IET and 2 PYT lines while the rest of the crosses involved IT99K-573-1-1 as a male parent. Five of the female lines were used twice, that is, they received pollen from both males. Overall, 222 parents were used to create 225 unique cross combinations that gave rise to a total of 1,436 F_1_. The F_1_ plants were technically half-sibs given that the progenies share two sister lines as their male parents but with different female parents. Sources of parental lines and statistics of the crossing design is provided in Table 1.

**TABLE 1:** Source of parental lines and the statistics of the crossing structure.

Source of lines	No. of lines	Lines used twice	No. crossed with IT97K-573-1-1	No. crossed with IT97K-573-2-1	No. of cross combinations	No. of F_1_
IET	89	3	77	15	92	428
PYT	31	2	31	2	33	214
AYT	85	0	85	0	85	637
Landraces	15	0	15	0	15	157
Total	220	5	208	17	225	1,436

*Total number of Male and Female Parents = (220 Females + 2 Males) = 222 parental lines. IET, Initial Evaluation Trial; PYT, Preliminary Yield Trial; AYT, Advanced Yield Trial.*

### Emasculation and Pollination

**FIGURE 1:**
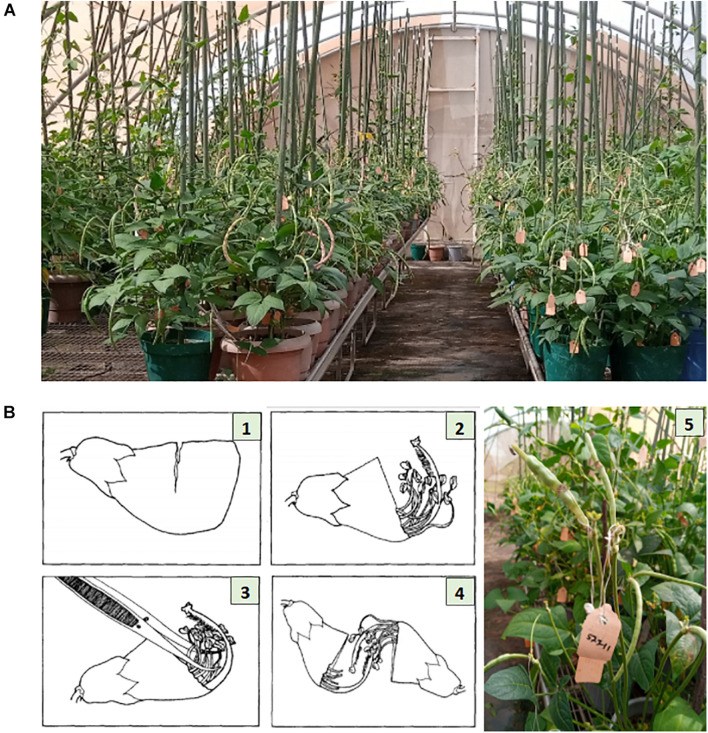
Illustration of cowpea crossing procedure. Panel **(A)** shows the cowpea crossing block with genotypes growing in pots placed on crossing benches in a screen house at IITA research station, Kano Nigeria. Panel **(B)** depicts the crossing steps: (1) Cut about two-thirds the width of the unopened bud. (2) Gently tear off the cut segment exposing the anther sacs. (3) Remove all anther sacs with forceps. (4) Pollinate the emasculated bud. (5) Fix a tag beneath the pollinated bud, listing the cross and the date of pollination. Photo source: Illustration 1–4 is adopted from Myers (1996), Photo **(A,B)**-5 were taken from IITA cowpea crossing block, Kano Nigeria.

Parental lines were planted in pots of size 24 cm (height) × 25.4 cm (diameter), three-quarter filled with sterilized topsoil, and placed on the crossing benches in the screenhouse at IITA research station in Kano (Figure 1A). Each pot planted with three seeds of the same cowpea line and after emergence the plants were well-managed through application of fertilizer and regular spraying against insects. At flowering stage, cross pollination was conducted following the rapid hand emasculation technique developed by IITA (Myers, 1996). Pollination consists of removing the upper half of the petals starting with a partial cut opposite the stylar and staminal section, exposing the upper portion of the style, stigma, and stamens. This is followed by removal of the 10 pollen sacs with forceps. To reduce cross contamination, the forceps was sterilized with 75% ethanol in between hybridizations, and care was also taken to avoid touching the receptive green tipped stigmatic surface before pollinating. The emasculated flower buds were pollinated with freshly opened flower. The mass of pollen on the hairy-necked style was used as a brush to deposit pollen grains on the stigma. A small tag listing the cross and date was affixed to the pedicel beneath the pollinated bud. A summary of the steps followed during emasculation and pollination have been provided in Figure 1B. The developing pods following cross pollination were well-maintained through regular watering of the plants, fertilizer application, and protection against insects. At maturity the tagged pods were harvested, threshed, and resulting F_1_ seeds stored.

### Leaf Tissue Sampling for DNA Analysis

F_1_ seeds from each of the 225 crosses (or populations) were planted in the screenhouse in new pots and placed on the crossing benches. At 2 weeks after planting, a young trifoliate leaf from each F_1_ plant was sampled for DNA analysis. The sampling was done according to the required procedure by Intertek-Agritech laboratory (Intertek-Agritech, 2016). First, unique sample identifications (UIDs) were generated from the EiB galaxy website^1^ and these were used to track the samples throughout from the time of sampling up to genotyping. The F_1_ plants to be sampled were labeled and a single hole-puncher (6.0 mm diameter) was used to punch and collect two leaf discs per sample from young and healthy newly developed trifoliate leaf. The punched leaf discs from each sample were transferred with forceps directly into a single well of 96-well plates (1.2 ml AbGene Storage Plate, number AB0564, Thermo Fisher Scientific). Wells H11 and H12 of the sampling plates were left blank as control wells according to Intertek-Agritech lab standard protocol. The forceps were cleaned before and after placing each sample in a well to avoid cross contamination. The sample plates were later placed in a box containing silica gel and allowed to dry slowly for 2 weeks. The 96-well sample plates were sealed with sealing mats (AB0674, Thermo Fisher Scientific), wrapped in plastic bags, secured firmly and shipped for DNA isolation and KASP genotyping to Intertek-Agritech laboratory in Sweden.^2^

### DNA Isolation and Genotyping

High throughput DNA extraction was achieved with the aid of robotics at the Intertek-Agritech lab. Genotyping was done based on KASP assay for 17 cowpea QC SNP markers. QC SNP markers for hybridity testing were selected by leveraging a previous study where cowpea parental accessions were genotyped with a 1,200 SNP Illumina GoldenGate assay (Muchero et al., 2009a). Marker selection considered a set of criteria to establish a SNP panel that could serve a broad range of cowpea breeding pools. In order to set up an assay relevant for different cowpea breeding pipelines, each SNP was considered for neutrality such that the probability of genotype calls for allele 1 and allele 2 across a diverse set of cowpea accessions were near 50%. If SNP calls were not neutral, i.e., calling allele 1 >75% of the time (or calling allele 2 > 75% of the time), those markers were considered biased and not considered for the QC SNP markers. In addition, SNPs were also selected for low percent heterozygosity (<4% heterozygous calls). Finally, with exception of linkage groups 1, 2, and 8 where one marker each was found to meet the technical validation criteria, two markers were selected per linkage group and to the extent possible, spread across the genetic map capturing regions at opposite ends of the centromere in order to maximize ability to capture recombination and probable crossovers. Once markers were selected, LGC genomics furthered the assay development leveraging the KASP marker system for cowpea genotyping (Boukar et al., 2016). The list of SNP markers and their genomic positions have been provided in Table 2 and are also accessible through excellence in breeding (EiB) platform.^3^

**TABLE 2:** Quality control (QC) Kompetitive Allele-Specific PCR (KASP) markers used for hybridity test.

SNP ID	Chr.	Position (ref. v1.1.)	Allele 1	Allele 2	Source
snpVU0007	1	36773526–36773649	T	C	IITA
snpVU0011	2	22941996–22942128	T	C	IITA
snpVU0018	4	16415787–16415919	A	G	IITA
snpVU0019	4	24230438–24230570	T	G	IITA
snpVU0001	5	399824–399956	C	G	IITA
snpVU0002	5	43326556–43327417	A	G	IITA
snpVU0009	6	30511313–30511445	A	C	IITA
snpVU0010	6	34246871–34247003	T	G	IITA
snpVU0003	7	4914544–491665	T	C	IITA
snpVU0004	7	39680298–39680430	T	C	IITA
snpVU0008	8	34271840–34271972	A	G	IITA
snpVU0012	9	29111205–29111337	A	C	IITA
snpVU0013	9	37010557–37010817	A	T	IITA
snpVU0016	10	37900312–37900440	A	G	IITA
snpVU0017	10	967432–967564	C	G	IITA
snpVU0014	11	34083600–34083732	A	G	IITA
snpVU0015	11	12936036–12936168	T	C	IITA

*Source: https://excellenceinbreeding.org/sites/default/files/u5/KASP%20markers%20for%20Cowpea.xlsx. Chr., Chromosome.*

### Statistical Analysis

#### Data Quality and Estimates of Genetic Parameters

Genotyping data from Intertek-Agritech lab were examined for completeness and quality using SNPviewer genotyping software developed by BIOSEARCH TECHNOLOGIES/LGC genomics.^4^ The software allows genotyping data to be visualized as a cluster plot on a plate-by-plate basis, revealing the clusters that group the similarity in genotyped allele calls. We were therefore able to evaluate and confirm the genotype call of each sample relative to the genotyping data obtained. A representative cluster plot for the first four SNPs in plate one is presented in Supplementary Figure 1-1. Genotyping data was converted into a HapMap file format and subjected to QC analysis in TASSEL 5 (Bradbury et al., 2007) by filtering for missingness and minor allele frequency (MnAF). Summary statistics for extent of missing data, overall heterozygosity and average MnAF for the filtered data was generated using the “Geno Summary” function of the data panel in TASSEL and output exported to R for graphical visualizations. Overall, average polymorphism information content (PIC) for markers using data on both F_1_ and their parents was estimated from the allele frequencies according to Anderson et al. (1993) as:


$${\text{PIC}}_{j}=1-\sum_{i=1}^{n}{\text{p}}_{i}^{2}$$

where; *i* = the *i*^*th*^ allele of the *j*^*th*^ marker, *n* = the number of alleles at the j^*th*^ marker, and *p* = allele frequency.

#### Analysis of Polymorphism and Marker Efficiency

To determine polymorphism of markers between the parents of the F_1_s, the genotyping data were sorted in Microsoft Excel to bring the 225 cross combinations together. The SNP markers that differentiated between each pair of parents in a cross were identified and the numbers recorded. The proportion of polymorphic markers in each cross were obtained as a ratio of the number of polymorphic markers to the total number of markers used, expressed as:


$$\%\text{Parental marker Polymorphism}=\left(\frac{\text{Pm}}{\text{Tm}}\right)*100$$

where; *Pm* is the number of polymorphic markers per pair of parents and *Tm* is the total number of markers used to genotype the pairs of parents.

To assess the efficiency of each marker in differentiating between the parents of a cross, each marker was evaluated for polymorphism across all the 225 parental pairs using a score of 1 = Polymorphic, 0 = Not polymorphic, NA = missing data (Supplementary File 1-1). The scores were then used to compute the performance of each marker by counting the number of times a marker was polymorphic in the pool of 225 parental pairs. Marker efficiency was computed as:


$$\text{Marker}\,\mathrm{efficiency}=\left(\frac{\text{fm}}{\text{Tc}}\right)*100$$

where; *fm* is the frequency of marker polymorphism among parental pairs (i.e., the number of times a marker is polymorphic across 225 parental pairs), *Tc* is the total number of parental combinations (excluding pairs with missing SNP calls). The computed marker efficiency was used to visualize the performance of each marker using a bar plot generated in R studio version 3.6.2 (R Core Team, 2019).

#### Analysis of Genetic Relationships

The KASP fingerprints of the 222 parents were used to evaluate genetic relatedness among the parents. The data was restricted to parents and a genetic dissimilarity analysis was conducted in TASSEL 5. For this analysis, parental lines with more than 10% of missing SNP data were removed, leaving 184 lines for diversity analysis. A second data set merging SNP data for both the F_1_ and parents was used to assess the relationships among the progenies and their parents. Genetic relationship trees were created using Neighbor-Joining clustering method (Nei and Saitou, 1987) and visualized using Archaeopteryx tree viewer in TASSEL 5. Principal component analysis (PCA) was conducted using the merged data to assess the contributions of markers and the relationship between the progenies and their parents. PCA values were generated using the *prcomp()* function in base R. A scree plot and cumulative variance plot depicting amount of variance explained by PCs were generated using *screplot()* and *cumsum()* functions of base R, respectively. PCA visualizations for marker contributions and parents vs. F_1_ scatters were performed utilizing *factoextra* R package (Kassambara and Mundt, 2020).

#### Hybridity Assessment

Initially, a general comparison was made between the F_1_s and the parents based on the proportion of heterozygosity obtained from TASSEL 5. The distributions of heterozygosity level between F_1_s and the parents were visualized using bar charts and box plots in R. Determination of the hybrid status of all F_1_s were based on the number of polymorphic loci that were heterozygous. Therefore, for each F_1_ sample, counts of SNPs detecting it as a true hybrid (heterozygous) were made and expressed as a ratio of the total number of markers that were polymorphic between its parents. Hybridity status of each F_1_ plant was computed as:


$$\text{Hybridity}=\left(\frac{{\text{L}}_{\mathrm{het}}}{\text{Pm}}\right)*100$$

where; *L*_het_ is the number of polymorphic SNPs detecting an F_1_ as heterozygous (true hybrid), *Pm* is the total number of SNPs that are polymorphic between the parents of a particular F_1_. Samples scoring <20% hybridity were regarded as untrue F_1_ (self). A pie chart was used to summarize the hybridity of the F_1_ samples into four categories: (i) successful (these are true F_1_s with hybridity scores of more than 20%), (ii) failed (these are untrue F_1_s which are products of self-pollination), (iii) undetermined due to missing data, and (iv) undetermined due to lack of polymorphism between the parents. In addition, a summary of hybridity success rate per population/cross was obtained a ratio of the number of true F_1_ plants in a cross divided by the total number of F_1_ plants in that cross.

## Results

### Marker Properties and Efficiencies

**FIGURE 2:**
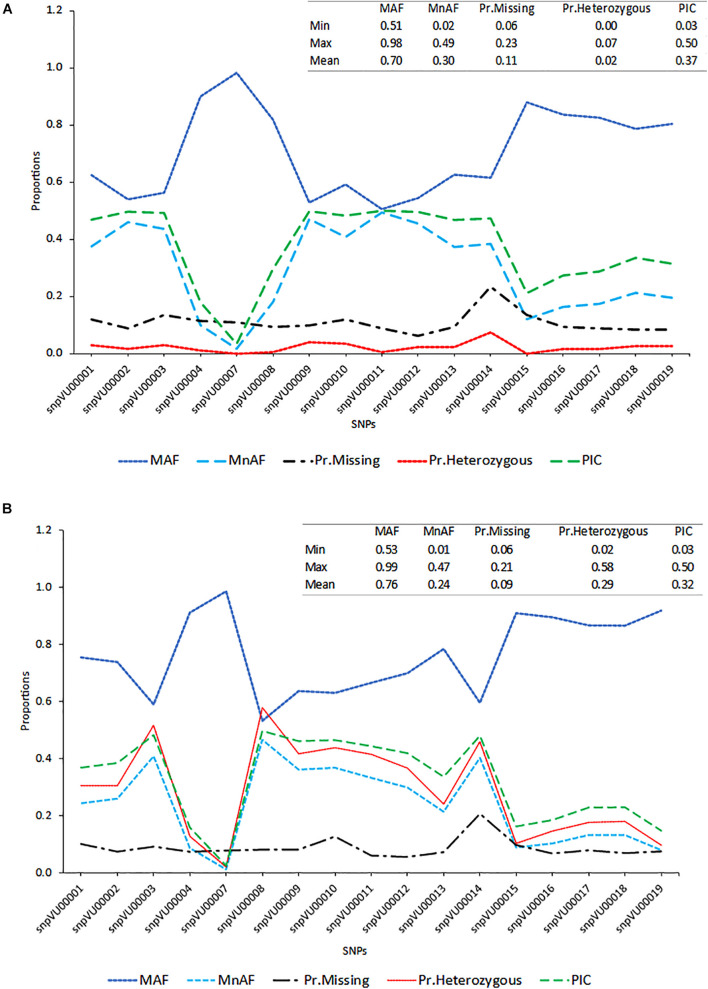
Distribution of major allele frequency (MAF), minor allele frequency (MnAF), proportion of locus heterozygosity (Pr.Heterozygous), proportion of missing data (Pr.Missing), and polymorphism information content (PIC). **(A)** Line graph based on combined data of both F_1_s and their parents. **(B)** Line graph based on data for parents alone. Inserts on the top right-hand corner of both graphs depicts summary statistics (Minimum, Maximum, and Mean values) of the estimated parameters.

To evaluate the accuracies with which the hybridity of the progenies were measured, the allele frequencies of SNP markers, locus heterozygosity (proportion of individuals identified to be heterozygous at specific marker locus), polymorphism information content (PIC) and the extent of missingness in the entire dataset were examined and the results are presented in Figure 2. The SNP markers showed variation in the genetic estimates, with s*npVU00007* having the lowest values for MnAF, PIC, and proportion of heterozygous loci among both F_1_s and the parents, while s*npVU00008* had the highest values. When both F_1_ and their parents were considered together, the average major allele frequency (MAF), MnAF, PIC, and heterozygosity were 0.76, 0.24, 0.29, and 0.32, respectively (Figure 2A). The MAF among F_1_ and parents ranged from 0.53 to 0.99, while MnAF varied from 0.01 to 0.4. Proportion of heterozygous loci among F_1_ and parents ranged from 0.02 to 0.58 and PIC ranged from 0.03 to 0.5. Similar values were observed when parents were considered alone except for proportion of heterozygous loci, where low proportions ranging from 0.0 to 0.7 and a mean of 0.02 were recorded (Figure 2B). In both cases, the patterns of MnAF, PIC, and heterozygosity per marker locus were similar and portrayed a sharp contrast with MAF. That is, the loci that showed high MnAF, PIC, and heterozygosity had low proportions of major allele frequencies and vice versa. These genetic properties provided a picture of which markers were more informative and could therefore accurately differentiate between parents and identify the true hybrids.

**FIGURE 3:**
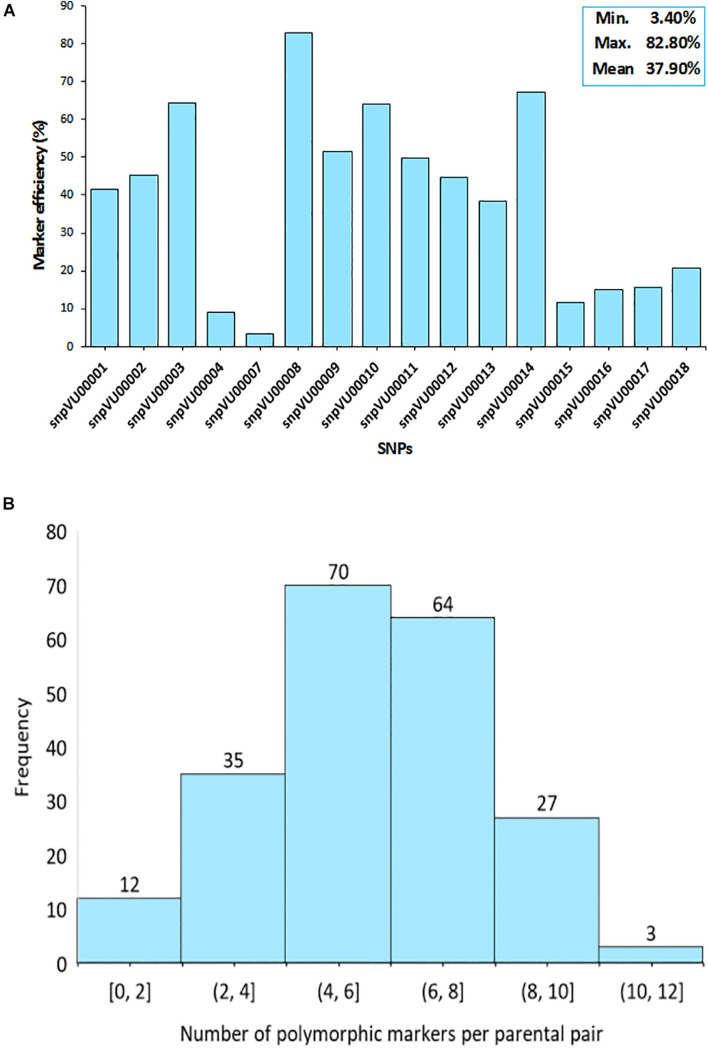
Informativeness of markers in differentiating parental pairs. **(A)** Bar chart shows the efficiency of each single nucleotide polymorphism (SNP) marker in distinguishing between pairs of parents. Insert on top right-hand corner is the summary statistics for marker efficiency indicating the Minimum, Maximum, and the Mean values. **(B)** Histogram depicts the distribution of polymorphism among pairs of parents as a measure of marker efficiency. Percentage of polymorphic markers in a cross is shown on the *x*-axis while the number of crosses is on the *y*-axis.

To further assess the effectiveness of the markers, the efficiencies were computed based on their ability to distinguish between the parents involved in a specific cross combination. The SNP markers displayed variable levels of polymorphisms between the parent combinations (Figure 3). The analysis revealed that s*npVU00008* was polymorphic across several pairs of parents and therefore, it was the best marker with 82.2% efficiency while s*npVU00007* was the least polymorphic and the least efficient marker (Figure 3A). The overall mean efficiency of markers in distinguishing between different pairs of parents was 37.90% with a range of 3.40–82.80%.

Consequently, there were some pairs of parents in which the level of marker polymorphisms was very low. This is depicted by the histogram in Figure 3B, where about 12 pairs of parents on the left tail of the distribution had between 0 and 2 informative markers. The number of informative markers in the rest of cross combinations ranged from 4 to 12 markers, with 4–6 informative markers being the most frequent. Marker polymorphism scores reflecting the number and percentage of polymorphic markers for each cross combination is presented in Supplementary File 1-1. The affected parental combinations with zero parental marker polymorphism included 2130-1x573-1-1 and 2091-2x573-2-1.

### Parental Fingerprints and Genetic Relationships

**FIGURE 4:**
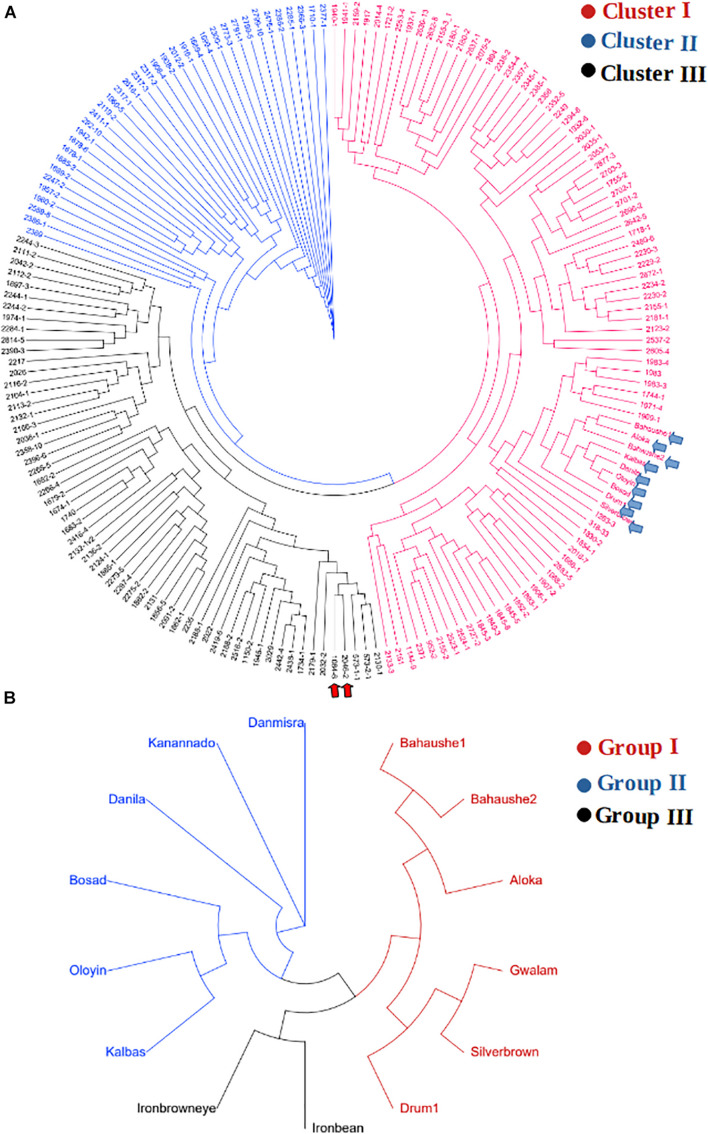
Relationship among parents that were used to derive the F_1_ progenies. **(A)** Cladogram showing grouping of parents into three clusters. The two elite varieties IT99K-573-1-1 and IT99K-573-2-1 were used as males and are emphasized in panel **(A)** by the red arrows, while cluster harboring the landraces are shown by the blue arrows. **(B)** Cladogram of landraces only, showing diversity among them despite being grouped together in Cluster I of panel **(A)**.

The KASP-based SNP fingerprints of all 222 parental cowpea lines is presented in Supplementary File 1-2. A total of 16 parents (7%) had unreliable fingerprints due to missing SNP information of more than 55%, while the KASP fingerprints of 209 other parents were of high quality, the profiles of which are suitable for future identity of these parents. Based on these fingerprints, the parents were found to display variable genetic relationships. Neighbor-Joining clustering algorithm divided the parents into three major groups (Figure 4A). A considerable diversity was still detectable within each of the sub-clusters. Cluster I harbored the highest number of parental lines including the landraces and was the most diverse cluster. The two male parents IT99K-573-1-1 and IT99K-573-2-1 occupied Cluster III and were close together in the same tree branch. Interestingly, all the landraces showed close genetic relationships and they occupied the same tree branch in Cluster I. However, when the landraces were considered alone, the diversity among them were noticeable (Figure 4B). The landraces formed three sub-groups with Bahaushe1, Bahaushe1, Aloka, Gwalam, silver brown, and Drum1 being in Group I, while Danmisra, Kanannado, Danila, Bosad, Oloyin, and Kalbas were in Group II, and Iron bean and Iron brown eye forming in Group III. Pairwise Euclidean genetic distances between 184 parents that had sufficient SNP calls ranged from 0 to 3.4 with a mean distance of 2.40 (Supplementary File 1-3). When we considered the relationships between 1 of the male parents IT99K-573-1-1 and the females, 19 pairs were found to have genetic distances below 2.0. Considering all the pairwise comparisons, a total of 183 parental pairs were identified to have genetic distances of <2.0 (Supplementary File 1-3).

**FIGURE 5:**
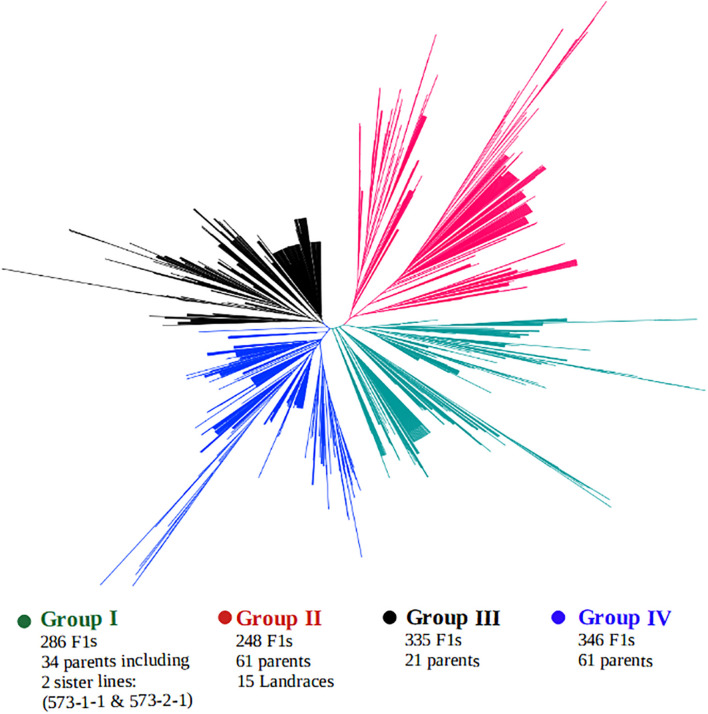
Clustering of F_1_ hybrids and the parents using 17 KASP-based single nucleotide polymorphism (SNP) markers. Four sub-groups were formed with parents distributed across all the groups. Group II contained the local varieties while Group I harbored the two male parents IT99K-573-1-1 and IT99K-573-2-1.

A clustering of both parents and the F_1_ hybrids based on SNP markers is presented in Figure 5. The genotypes were grouped into four main clusters, hereafter, referred to as the “Group I,” “Group II,” “Group III,” and “Group IV,” and the parents were clearly divergent from each other, as they are distributed throughout the four clusters. That is, the parents did not form any unique or a separate cluster of their own but were spread across all the four groups. Crosses involving the landraces were mostly clustered together with their parents in Group II. This group contained over 248 F_1_ progenies, of which 200 F_1_s carried alleles from a local varietal parent. Cluster analysis also revealed that over 286 F_1_ plants shared the same main cluster (Group I) with the 2 male parents IT99K-573-1-1 and IT99K-573-2-1. Groups III and IV had the highest number of progenies with 335 and 346 F_1_s, respectively.

**FIGURE 6:**
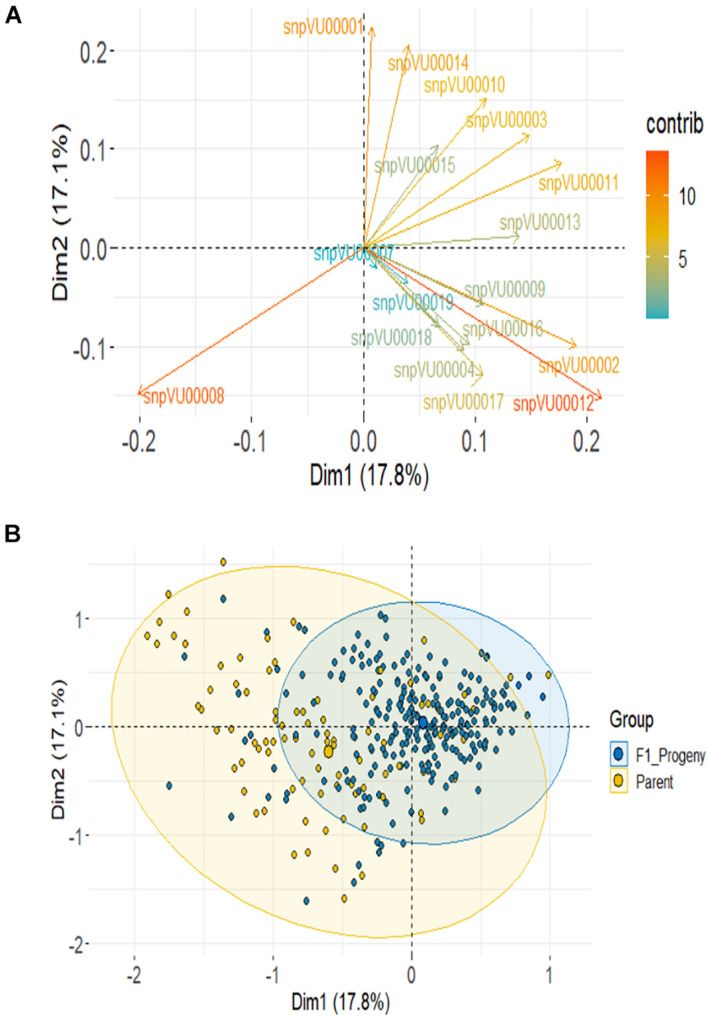
Principal component analysis (PCA) of F_1_ and the parents. **(A)** Contribution of each KAP-based single nucleotide polymorphism (SNP) markers to variation among genotypes; color scale on the right side indicates level of contribution. **(B)** PCA scatter highlighting the F_1_ progenies and the parents.

Based on SNP data, PCA using the combined F_1_ and parents showed the first 2 PCs to account for about 35% variation and it required 10 PCs to account for over 90% of total variance (Supplementary Figure 1-2). About 14 markers (82%) contributed most to the observed variation with *snpVU00012* and *snpVU00008* being the highest contributors, while *snpVU00007* and *snpVU00019* were the least contributors (Figure 6A), a result that is analogous to the outcome of polymorphism and marker efficiency analysis in Figures 2, 3. The PCA results depicted a majority of the F_1_ progenies to be closer together while the parents were widely distributed such that the F_1_s were a sub-set of the parents (Figure 6B).

### Hybridity of F_1_ Progenies

**FIGURE 7:**
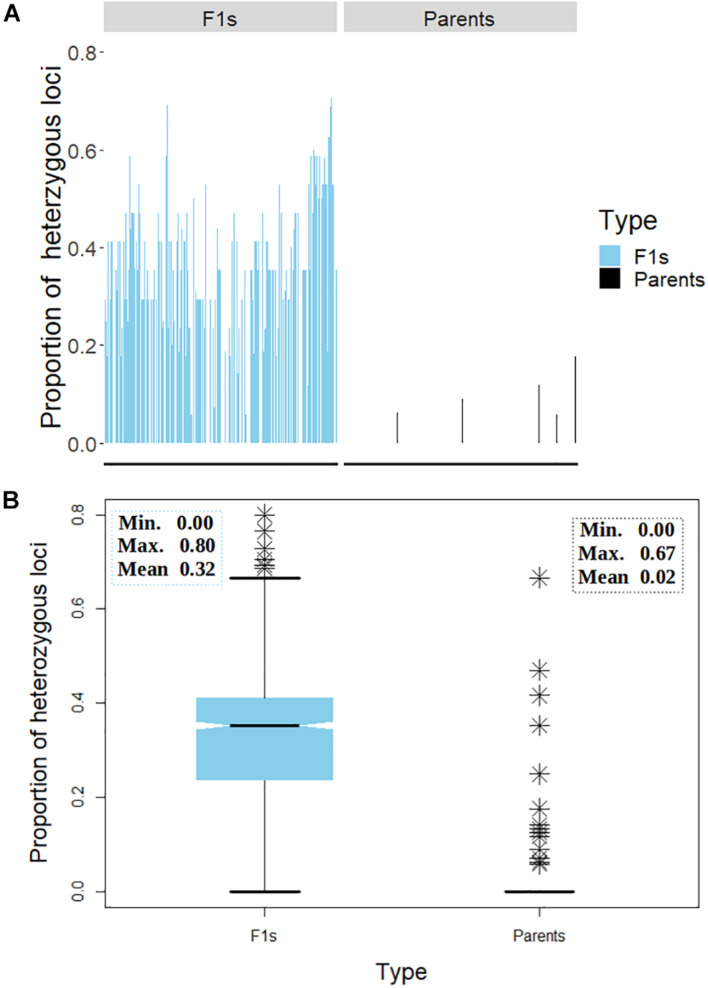
Distribution of heterozygosity between the F_1_ and their parents. **(A)** Bar chart displaying the proportion of heterozygous loci on the *y*-axis and the genotypes on the *x*-axis. **(B)** Box plot depicting the dispersion of heterozygosity between the F_1_ and the parents. Inserts in panel **(B)** depicts summary statistics; minimum, maximum, and mean heterozygosity among F_1_ and the parents, respectively. The symbol ^∗^ represents genotypes (both parents and F1s) with high proportion of heterozygous loci.

A general comparison of the proportion of heterozygous loci as a measure of hybridity of F_1_ progenies revealed a clear contrast between the F_1_ hybrids and their parents, with the F_1_ hybrids showing an overall higher heterozygosity than the parents as would be expected (Figure 7). However, there were few instances depicted in the bar plot (Figure 7A) and the boxplot (Figure 7B) where some F_1_ hybrids and parents displayed low and high proportions of heterozygous loci, respectively. In fact, 13 (6%) parental lines had more than 10% level of heterozygosity while 177 (79%) parents were 100% homozygous (Table 3). Of the 13 heterozygous parents 5 were recycled from IET stage, one from PYT, five from Advance Yield Trial (AYT), and two were landraces. The proportion of heterozygosity among F_1_ progenies ranged from 0 to 0.8 with a mean of 0.32, while for parents it ranged from 0 to 0.67 with a mean of 0.02 (Figure 7B).

**TABLE 3:** Summary level of heterozygosity among parents and F_1_ progenies.

Category	No. of parents	% of parent
**Among parents**		
Parents with 0% heterozygosity	177	80
Parents with 6–10% heterozygosity	5	2
Parents with >10% heterozygosity	13	6
Filtered out due to missing data	27	12
Total	222	100

**Category**	**No. of F_1_s**	**% of F_1_s**

**Among F_1_s**		
F_1_s with 0% heterozygosity	205	14
F_1_s with 6–10% heterozygosity	28	2
F_1_s with >10% heterozygosity	1,103	77
Filtered out due to missing data and non-polymorphic markers	100	7
Total	1,436	100

**FIGURE 8:**
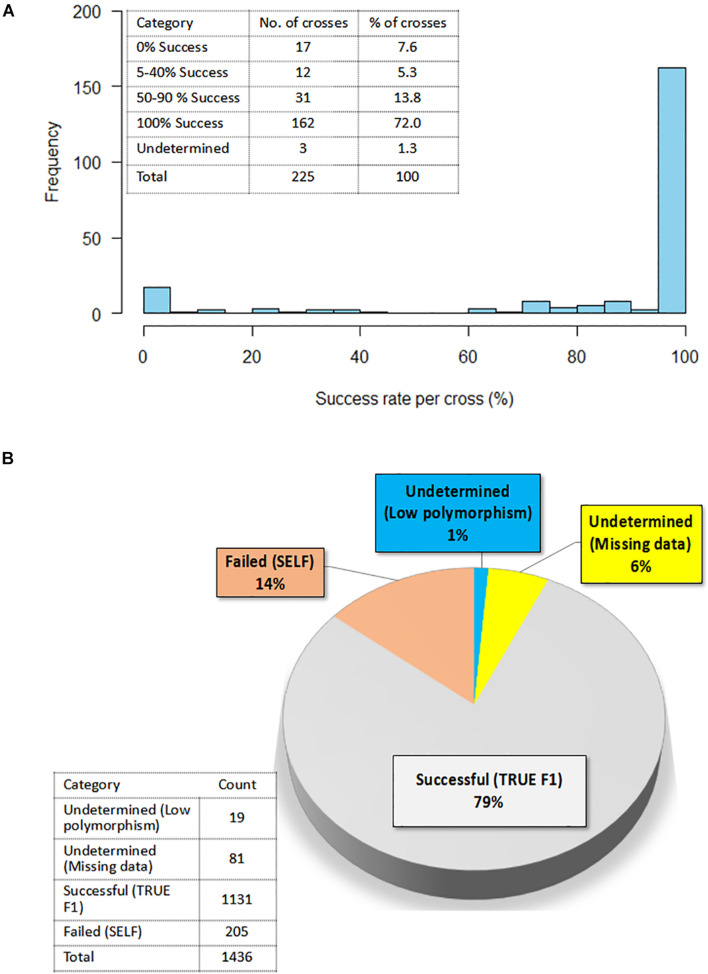
Confirmation of hybridity. **(A)** Histogram showing the distribution of success rate for true F_1_ hybrids in 225 crosses; Insert on the top left shows the exact number of crosses and a corresponding percentage in each success category **(B)** Pie chart depicting percentage of true F_1_s out of 1,436 F_1_ progenies as determined by the level of heterozygosity of F_1_ progenies for the polymorphic SNP markers; Insert on the left-hand bottom corner reflects the actual numbers of F_1_ progenies in each category.

A summary of hybridity success rate based only on polymorphic markers between parents is presented in Figure 8. The distribution in Figure 8A depicted a general shift toward high success rate with 72% of the crosses attaining 100% hybridity success, 19% had intermediate success rates, 8% had 0% success (that is, none of the purported F_1_s in these crosses were true hybrids), and 1% had undetermined hybridity due to lack of polymorphism and missing data. When we considered all the 1,436 F_1_ progenies individually and computed the hybridity success rate, the results identified 1,131 (79%) progenies to be true F_1_ hybrids (successful crosses) (Figure 8B). These progenies were also detected as being heterozygous at more than 5% of polymorphic loci (Table 3). However, 205 (14%) putative progenies turned out to the products of self-pollination, while the hybrid status of 1 and 6% of the progenies could not be determined due to lack of polymorphism and missing data, respectively (Figure 8B and Table 3).

## Discussion

The demands of mankind for food, feed and various agricultural products have been increasing. To develop products that meet this increasing demand, breeders are challenged to accelerate genetic gain continuously, at ever higher rates, while they bridge the gaps that remain between the yield potential in breeders demonstration trials and the actual yield in farmers’ fields (Xu et al., 2017). The use of molecular markers has been demonstrated to play key roles in breeding optimization to attain enhanced genetic gain, mainly, through improved precision and reduction in time through more rapid and efficient breeding cycles (Perez-de-Castro et al., 2012; Sussumu Sakiyama et al., 2014). The past 15 years has seen key advances in development of cowpea genetics and genomics resources (Singh, 2007; Muchero et al., 2009a; Boukar et al., 2016, 2018; Munoz-Amatriain et al., 2016; Muñoz-Amatriaín et al., 2017; Fatokun et al., 2018; Huynh et al., 2018; Lonardi et al., 2019). This has facilitated the discovery of molecular markers for traits discovery and deployment in the breeding programs. For example, a number of studies have identified quantitative traits loci (QTLs) associated with resistance to biotic and abiotic stresses in cowpea (Muchero et al., 2009b; Agbicodo et al., 2010; Lucas et al., 2013; Huynh et al., 2015; Benoit et al., 2016; Essem et al., 2019; Miesho et al., 2019). However, although the application of molecular markers in hybridity testing and hybrid purity assessment have been demonstrated in several other crops (Patella et al., 2019a; Kishor et al., 2020; Osei et al., 2020), there is no documentation of its deployment in cowpea. Our work, therefore, is intended to integrate SNP markers as a QC mechanism against the unintended self-pollination or cross contamination following hybridization. This study has demonstrated that the strategy is also effective in cowpea. This is of paramount importance considering the significance of cowpea in food and nutrition (Kebede and Bekeko, 2020) and the need for constant and rapid development of new varieties. Released varieties of cowpea are often pure lines given its autogamous nature. Manual pollination between homozygous parents carrying agronomic traits of interest is, therefore, required to introduce genetic variability required for improved variety development. Despite the efficiency of hand emasculation method developed in the past (Myers, 1996), it remains difficult to rapidly differentiate true F_1_ seeds from self-pollinated ones in cowpea and yet this is a crucial step in the breeding process. The use of molecular assays to quickly and accurately screen progeny makes it possible to overcome some of the potentially frustrating occurrences in conventional breeding. Our KASP-based SNP markers have facilitated routine use of low-density fingerprinting of cowpea parental lines and allowed accurate detection of hybrids amongst their progenies.

This study began by assessing the informativeness of 17 QC cowpea SNP markers based on allele frequencies and PIC. The minor allele frequencies refer to the proportion of the second-most-common alleles at a genetic locus in a population and play a key role in determining allele diversity and heritability in the population (Dussault and Boulding, 2018; Hernandez et al., 2019). Dussault and Boulding (2018) reported that markers with high minor allele frequencies have high resolving power in distinguishing between individuals. PIC on the other hand, measures the ability of a marker to detect polymorphisms, hence, it determines the quality of a molecular marker for genetic analysis (Serrote et al., 2020). Results from our study revealed that 16 out of the 17 (94%) markers had minor allele frequencies of >0.05 and PIC above 0.2. Average level of heterozygosity was 0.29 when both parents and F_1_ progenies were considered together but this measure dropped drastically to a mean of 0.02 when parents were considered alone, which suggested that the SNPs had the power to distinguish heterozygotes from homozygotes. Overall, the estimates of these marker properties implied that the 17 SNP marker panel used were informative, that is, they were capable of distinguishing between parents, henceforth, identify true hybrids. These outcomes were supported by the estimates of marker efficiency which revealed only one marker (s*npVU00007*) with the lowest marker efficiency of about 3.4%.

We continued to assemble SNP fingerprints of parental lines and used them to dissect the genetic relationships among parents and the F_1_ progenies to discern which crosses benefited from maximum diversity between the parents. Analysis of genetic relatedness among plant germplasm plays a key role in plant breeding as it allows effective characterization of parental lines for hybridization (Govindaraj et al., 2015; Mafakheri et al., 2017; Khanna, 2018; Nkhoma et al., 2020). In the present study, genetic similarity analysis placed the parental lines into three groups, with a modest amount of variation still detectable within the groups. The landraces were grouped together in Cluster I, however, when considered alone, a considerable diversity was detectable among them and they were clustered into three sub-groups. The clustering of the landraces together when the all lines were considered is likely due to the fact, they are quite unrelated to the other parental lines, and as such the cladogram placed them in the same sub-group. Interestingly, a number of the parental lines shared the same cluster with the landraces, and therefore, they potentially carry some beneficial adaptation traits. As expected, the two male parents IT99K-573-1-1 and IT99K-573-2-1, showed 100% similarity and were grouped together with several other parents in the same cluster. Given that the rest of the parental lines were females, hybridizations between all closely related females and the two sister male lines is likely not beneficial. The parental similarity analysis, therefore, allowed us to identify progenies from crosses that involved closely related parents. Most of the female parents were selected from IET, PYT, and AYT based on their yield potentials and quickly recycled into the hybridization program. However, it was expected that some of these parents would be closely related since they shared common pedigrees. The fact that crosses were made prior to genetic similarity analysis, results from the present study provided a QC tool for early discarding of crosses with closely related parents. Representative crosses can be selected based on parental clustering and advanced in the breeding program. While comparing the F_1_ and their parents, the first two principal components revealed a considerable overlap between the F_1_ and parents. However, the F_1_ progenies appeared to be more closely related to each other while the parents were highly divergent. This outcome is expected given that all the F_1_ progenies were technically half-sibs, having received a common set of alleles from the two sister lines (IT99K-573-1-1 and IT99K-573-2-1). The expected variation for effective selection at later generations, therefore, comes solely from the diversity among the female parents. It is important therefore, to sort the crosses to be advanced based on relationships among parents.

A final focus was made on the molecular assessment of hybridity of the F_1_ plants derived from multi-parental crosses. Molecular determination of hybridity has been successfully demonstrated in various crops; tomato (Osei et al., 2020), maize (Salgado et al., 2006), lettuce (Patella et al., 2019a), Capsicum (Mongkolporn et al., 2004), rice (Yashitola et al., 2002), cotton (Dongre and Parkhi, 2005), cassava (Vincent et al., 2014), and peanut (Gomez et al., 2008). Apart from that on tomato, these studies deployed microsatellite markers for hybridity testing as a QC to determine true F_1_ for advancement. The present study, utilizing 17 KASP-based SNP markers, determined the homozygosity of parental lines and successfully identified true F_1_s. We found that 6% of the parents were unstable (heterozygous) at 2–8 loci, however, their inclusion as parents in hybridization was based on their yield potential and several other farmer-preferred attributes. Most of the parents detected as heterozygous were recycled at IET (F_5_ generation), PYT (F_6_ generation), and AYT (F_7_ generation) stages, while two of them were landraces. Heterozygosity observed among parents could be attributed to residual heterozygosity, outcrossing and heterogeneity (Plaschke et al., 1995; Fatokun and Ng, 2007; Gioi et al., 2012). To distinguish between F_1_ individuals resulting from cross-pollination and those resulting from self-pollination, we first examined the informative loci among the parental lines used in the crosses. Only polymorphic loci between pairs of parents were considered informative and used in hybridity assessment. Among the cross combinations, our analysis showed a range of 0–11 informative markers out of the 17 SNPs, 4–6 being the most frequent (70 parental combinations). Overall, 94% of the parental combinations had more than two informative markers, allowing for the authentication of putative hybrids from these cross combinations. Patella et al. (2019a) reported that restricting this kind of analysis to the informative loci brings considerable savings in terms of time and costs. All in all, molecular assessment of hybridity was successful: Over 72% of the crosses yielded 100% true F_1_ progenies (100% success rate) while 8% had unsuccessful F_1_ offspring (Selfed progeny). In lettuce, a highly self-pollinated crop, 100% success rate was reported in only 30% of the crosses (Patella et al., 2019a). Considering all the 1,436 F_1_ plants developed through manual hybridizations and tested in the present study, a success rate of 79% was attained. In peanut, out of 179 putative F_1_s, 70% were detected by SSR marker to be true hybrids (Gomez et al., 2008). To our knowledge, the present study is the first report on hybridity testing using molecular makers in cowpea. The crossing success rates of 10–20% reported by Myers (1996) and 28.36% by Ajayi et al. (2020) in cowpea are not necessarily the percentage of true F_1_s obtained, but the percentage of hybridizations that survived mechanical handlings and other stresses during manual emasculation. Our study found that of the hybridizations that survived mechanical handling during hand emasculation, 14% were a product of self-pollination. We also found that the hybridity status of about 1% of the progenies could not be determined because all the 17 markers were not polymorphic between their parents. This lack of polymorphism was observed in about 12 parental pairs and this could be attributed to the genetic similarity among parents. In fact, when we checked the pedigrees of lines involved in these parental pairs, we found that 9 out of 12 had 573-1-1, 573-2-1, and IT97K-499-35 as 1 of their parents. Interestingly, all the three lines (IT99K-573-1-1, IT99K-573-2-1, and IT97K-499-35) share a common striga resistant parent IT93K-596-9-12 (Singh et al., 2006). The remaining three lines shared TVu450 genome, a landrace with unknown pedigree, as one of their parents. These observations provide a plausible reason for lack of polymorphism between these lines and 573-1-1/573-2-1 (the two sisters lines used in this study). The limitation of detecting differences among closely related lines could be minimized by increasing the number of markers for hybridity tests and using genetically diverse parents. Usually, 20–30 SNP markers that are carefully chosen to have good genome coverage is sufficient (PASSeL, 2019). However, different marker densities, some with lower numbers than what is employed in the present study have been used to successfully dissect hybridity and fingerprints in crop plants (Nandakumar et al., 2004; Romdhane et al., 2018; Josia et al., 2021). According to Josia et al. (2021), the common assumption that the use of a large number of markers results in higher accuracy should be considered carefully due to the fact that in most sequence-based marker systems, the levels of missing data can lead to wrong interpretation. Therefore, the selection of fewer markers with high and repeatable representation across samples is desired and is cost-effective. Chen et al. (2016) also noted that fewer markers with high expected heterozygosity, missing value of <20%, and observed heterozygosity of <6% are ideal markers for accurate QC genotyping. The present study used 17 informative SNPs and we included as control, known sister lines and landraces which are quite diverse from some of the elite breeding lines. The fact that these markers were able to consistently diagnose these differences and further delineate the F_1_s from the homozygous parents demonstrated the effectiveness of the 17 QC marker set for fingerprinting and hybridity testing of cowpea.

## Conclusion

The results of this study contribute to the body of knowledge about the advantages of integrating molecular markers as a QC step in a breeding program. We used KASP-based SNP markers in the first breeding phase which involves parental selection and identification of true hybrids. We demonstrated, firstly, the efficiency of SNP markers not only in detecting the best parental lines for crossing based on their observed homozygosity and genetic relatedness, but also in screening the resulting F_1_ progeny to distinguish between the offspring resulting from cross-pollination and those resulting from self-pollination. The markers identified true hybridity with 100% success in 72% of the populations. Overall, we detected 79% of the putative F_1_ plants to be true hybrids while 14% were selfed plants. Furthermore, given the ability of the SNP marker panel to detect heterozygosity or admixture in the parental lines, these markers can be utilized downstream the breeding scheme to assess the uniformity of potential candidate or pre-commercial cowpea lines. The fact that these sets of SNP markers are reproducible through the KASP assays, the genotype data from this study constitute a molecular fingerprint of the parents that would be utilized for future identity and purity assessment. The markers could also be used by seed companies to assess newly developed varieties for distinctiveness, uniformity and stability (DUS), which are the three key necessities for registering plant materials (Patella et al., 2019b).

## Data Availability Statement

The original contributions presented in the study are publicly available. This data can be found here: European Nucleotide Archive (https://www.ebi.ac.uk/ena/browser/home) database under accession number: PRJEB46458.

## Author Contributions

PO wrote the manuscript, organized samples for genotyping, and conducted the statistical analysis. CF, OB, AT, and SS revised the manuscript. OO, MA, IJ, and GB organized and coordinated pollination and tissue sampling for DNA analysis. OB and CF developed the populations and designed the study. All authors reviewed and approved the manuscript.

## Conflict of Interest

SS is employed by the company Bayer Research and Development Services LLC. The remaining authors declare that the research was conducted in the absence of any commercial or financial relationships that could be construed as a potential conflict of interest.

## Publisher’s Note

All claims expressed in this article are solely those of the authors and do not necessarily represent those of their affiliated organizations, or those of the publisher, the editors and the reviewers. Any product that may be evaluated in this article, or claim that may be made by its manufacturer, is not guaranteed or endorsed by the publisher.

## Footnotes

1
http://13.250.212.83

2
http://www.intertek.com/agriculture/agritech/

3
https://excellenceinbreeding.org/sites/default/files/u5/KASP%20markers%20for%20Cowpea.xlsx

4
https://www.biosearchtech.com/support/tools/genotyping-software/snpviewer
